# An Essay on Curvatures and Distortions of the Spine, and Some Other Morbid Derangements to Which It Is Subject

**Published:** 1822-11

**Authors:** R. W. Bampfield

**Affiliations:** One of the Surgeons to the Royal Metropolitan Infirmary, &c.


THE LONDON
### Medical and Physical Journal.

5 OF VOL. XLVIII.] NOVEMBER, 1822. [No. 285.

For many fortunate discoveries in medicine, and for the detection of numerous errors, the world is indebted to the rapid circulation of Monthly Journals; and there never existed any work, to which the Faculty, in Europe and America, were under deeper obligations, than to the Medical and Physical Journal of London, now forming a long, but an invaluable, series.—RUSH.

### ORIGINAL COMMUNICATIONS, SELECT OBSERVATIONS, &c.

ART. I.—*An Essay on Curvatures and Distortions of the Spine, and some other Morbid Derangements to which it is subject.* By R. W. BAMPFIELD, Esq. one of the Surgeons to the Royal Metropolitan Infirmary, &c.

> "What can we reason, but from what we know?"
> POPE'S *Essay on Man.*

THE sublime privilege of standing erect, with the eyes directed to the star-decked vault of heaven, has been exclusively granted to man, and confers on him peculiar advantages and dignity. To preserve to him this valuable attitude, the all-wise Creator has constructed the spine like a pyramid, differing, indeed, from the motionless piles of architecture, for its mechanism is inimitable: still, from its shape, it has been long compared to two pyramids with a common base; but the upper one, that is formed of the twenty-four true vertebræ, by which the several motions of the trunk of the body are performed, is the particular subject of our lucubrations and observation, because it is the seat of the diseases and deformities intended to be discussed and treated of in this essay.

It will not be necessary to enter into a minute anatomical description of the spinal column; it will be almost sufficient to observe, that it is formed of twenty-four joints, which are moveable, strongly conjoined, and, as a whole, admit of considerable flexion forwards, of a small degree backwards, and of some extent of lateral motion; but, by whatever causes a deflection is made from the straight or erect position, adequate moving powers are provided to restore it to its natural and noble attitude, as long as the primary form of the body is preserved entire, the muscles retain their contractile powers, and the different bones, of which the spinal column is formed, preserve their relative proportions. From the complicated mechanism of the spine, by which all its various parts are adapted with so much wisdom and order to the different purposes they were designed for, and its movements balanced with so much exactness and care to secure the structure from being deranged or displaced; from the strong muscles spread over the posterior part of the vertebræ, to prevent the column from falling into a permanent inflection forwards; from the disposition of the numerous bony processes, and the insertion of the ribs, which secure so many of the vertebræ from being displaced laterally and anteriorly; from the strength and number of the ligaments, which are unyielding, almost inelastic, and which connect the joints of the vertebræ with a firmness and power more than equal to protect them against all ordinary exigencies or ordinary force to be resisted, so that fractures of the bodies of the vertebræ are, beyond comparison, more numerous than dislocations:—from all these wise provisions of the Creator, and some minor ones not enumerated, it would, *a priori*, seem scarcely possible that distortions of the form and derangements of the order of this structure should so frequently present themselves; for, viewing the spinal pyramid as a piece of mechanism judiciously constructed and artfully secured, it might be inferred that no mechanical power but the wedge could permanently displace any of its parts; yet this deformity has existed, and has engaged the descriptive pen of poets and of surgeons, and its cure, alas! has baffled the attempts of preceding ages,—at least such a cure as generally restores the vertebræ to their natural condition, position, and powers of movement. Is the attainment of such a cure devoutly to be wished for? If it be, we intreat our efforts may be received with indulgence; and we hope our labours, if they merit it, will be fostered by encouragement: at all events, the results of our endeavours shall be faithfully made known to the public.

Curvatures, projections, and deformities of the spine, have been noticed by the best surgical writers of antiquity, as well as those of more modern date; and although, in our days, many surgeons of acknowledged talent and originality of mind have directed their attention to them, and contemplated them with an anxious desire to remedy those morbid derangements effectually, yet it must be admitted as a fact that, in the public institutions of Europe, the practice in those cases has not much improved since the days of that classical and experienced surgeon, Mr. Pott; while the principle on which the treatment is founded continues the same, and influences all; for the treatment is generally directed to the sole object of forming an anchylosis of the diseased or deranged vertebræ in the situation the distortion has placed them, by inducing the parts to accommodate themselves to the degree of projection or curvature already established. This principle, and the mode of cure to which it has led, necessarily leave the patients, in many cases, more or less deformed for life. Hence it is, the practice in those cases appears imperfect; whilst its results would seem to justify its abandonment, and sanction any cautious and promising experiments that reason should suggest.

That the defects of the *modus medendi* in general use may not be thought unjustly overcharged, it may be proper to state that the best elementary works on Surgery,—that the able prize Dissertation, the author of which of late was deservedly honoured by the medal of the Royal College of Surgeons, and the latest treatises on Diseases of the Spine, attribute its curvatures to carious bone, and consider the application of the caustic issues of Mr. Pott, and the observance of the horizontal position inculcated by the late Mr. Baynton, as the principal, if not the only, means necessary to their cure, by anchylosis.

Before entering at large on the subject, it seems requisite to premise definitions of some terms, that will be either new or employed in a more limited sense, and with a more precise meaning than heretofore.

Curvatures of the spine have been divided into two species, one of which has been termed "angular curvature," and the other "lateral curvature."* The first includes all the varieties of curvature, attended with projections from the natural axis of the vertebræ, either outwards or inwards; the second comprehends all those distorted or bent on either side. Our mathematical friends inform us the term "angular curvature" is a solecism in mathematics, and that the existence of such a curve or arc has not been demonstrated; it is, therefore, inferred it cannot convey any precise meaning, as an angle cannot be a curve, or a curve an angle, although every curve contains an angle, and that its use may be declined without impropriety.

All the distortions and projections of the spine we have witnessed may be divided into two species, one of which may still be denominated curvature of the spine, and the other the angular projection of the spine. Of the former there are three varieties, the first of which that will be noticed is the curvature outwards, which, for the sake of brevity, we propose to term an *excurvation* of the spine;† the second is the curvature inwards, which, in this essay, will be called an *incurvation;* the third is the curvature on either or both sides, which is, with propriety, already denominated the *lateral curvature.* The angular projection has not any varieties. Curvatures of the spine may take

* Lloyd on Scrofula.

† Excurvation is not a genuine English word: it is trusted, however, its use will be excused; for, as incurvation very aptly expressed one condition of the curved vertebræ, it was thought excurvation might be as fitly employed in contradistinction.

place in any portion of the spinal column; they, however, most commonly occur in the cervical and dorsal vertebræ. The angular projection generally takes place in the lumbar and lower dorsal vertebræ. A curvature of the spine forms a segment of a circle, the arc of which is more or less regular or perfect.

The line the vertebræ, or spinal column, ought to make, and does make in its natural undeformed state, will be called the *spinal line;* a term that will be nearly synonymous with that of axis of the vertebræ.

The angular projection of the spine forms the projecting line of an acute angle with the spinal line, the quantity of which varies in the degree the spinous processes project beyond their natural situation. The spinal line, the projecting line, and the line formed by the lower surface of the most projecting vertebra, form a triangle, of which we will consider the last, with its spinous process, to be the base.

Horizontal position is a term much used in treating of diseases of the vertebræ. It is evidently a vague and general term, as it only implies laying the body down on an horizontal plane, without reference to particular position. When the position proper in curvatures of the spine is alluded to by authors, horizontal position has generally been defined by them—laying on the back, either on a horizontal or (by some) an *inclined* plane. The advantages, however, of particular positions of parts, or of the whole of the body, in some operations of surgery, are so well known, and so highly estimated, (as, for instance, in reducing the different luxations of the head of the thigh-bone,) that they will obviously occur to every one; and as curvatures of the spinal pyramid take different directions, and form a variety of deflections from the straight line, so experience has fully convinced us that different positions of the body can be adapted to the different forms of curvature with striking and peculiar benefit. For the correct and clear comprehension, therefore, of the terms and operations mentioned in this essay, it is necessary to state that three variations of the horizontal position will be employed in the treatment:—The *dorsal* horizontal position, or laying on the back; the *facial* horizontal position, being the reverse of the former, or what, in common language, is called "lying on the face," of course in a line with the sternum and linea alba abdominis; the *lateral* horizontal position, or lying on either side. In all cases, the patients are supposed to be laying on a horizontal plane; as we hope, hereafter, to demonstrate very clearly the inconsistency of using the inclined plane in diseases of the vertebræ, from its being both a mathematical deviation from the horizontal position, approaching more or less to the perpendicular, and a violation or infringement of the received and approved principle on which the former has been generally adopted.

It is deemed right to distinctly avow, *in limine*, that the principle of cure by promoting and consolidating an anchylosis of the different vertebræ deranged, is, in this essay, entirely rejected *as a general principle* which should guide and direct the treatment of curvatures of the spine; neither can we subscribe to the truth of the statement of so many authors, that a cure without anchylosis cannot be accomplished; for our experience, as far as it extends, has sufficiently satisfied us that the body can be restored to its erect attitude, and the joints of the vertebræ to their position and powers of movement, in all cases, except only where the ulcerative process (whether produced by scrofula or other causes,) has already seized on the structure of the vertebral joints, and renders anchylosis desirable, as well as indispensably necessary to recovery in any shape. Hence it is that the principle of cure proposed and illustrated in this essay will be the very reverse of the former; and it will be laid down as a settled general principle or fixed indication, that the curative efforts, in curvatures of the spine, should be directed to the prevention of anchylosis as their primary object, feeling quite assured that success will be obtained, by the means to be hereafter detailed, in a great majority of cases; the exceptions being limited to that variety already mentioned, the occurrence of which, on an improved plan of treatment, will be still more rare than it really is. Although a late able writer affirms, "that the angular curvature is most to be dreaded, because it never can exist, in the smallest degree, without some destruction of bone,"* yet, as the angular curvature of Mr. Lloyd includes the incurvations, excurvations, and angular projections of the spine of this Essay, and as we have not as yet had sufficient reason to believe that ulceration of the bony parts of the vertebræ existed in any case we have treated, we still feel at liberty to assert and maintain the opinion, that curvatures of the spine, with ulceration of the bony parts, are comparatively rare.

In compliance with the wishes of some respected medical friends, a few cases of distortion of the spine will be detailed in this part of the Essay, by which some of the material of which the structure is to be built will, as it were, be presented for examination and judgment; and the reader, it is hoped, will be compensated for a departure from the systematic arrangement of describing symptoms, discussing causes, and inculcating doctrines and modes of treatment, in regular order, before the history of cases is given.

* Lloyd on Scrofula, p. 218.

### *Cases of Angular Projection of the Spine.*

CASE I.

Frances Parker, æt. four years, nine months, born of healthy parents, and one of six children, neither of whom presented the features of a scrofulous constitution, residing at No. 3, Slade's-place, Blandford-street, had been affected with the angular projection of the spine two years, when she was admitted a patient of the Mary-le-bone station of the Royal Metropolitan Infirmary. The projection had been formed in a gradual manner, one spinous process only having been first observed to protrude outwards; and, as this projection enlarged and increased, it became evident that other vertebræ above it, in the progressive course of time, were drawn out also. The projection comprehends three lower dorsal and four upper lumbar vertebræ. When the patient stands erect, the projection of the spinous process of the fourth lumbar vertebra is so much beyond that of the fifth, that it appears as if, at this point, the upper part of the spinal column were about to fall off from the part below it. From the spinous process of the ninth dorsal vertebra, which occupies its natural situation, the spinous processes of the seven vertebræ below it gradually incline outwards, till the line reaches the extremity of the spinous process of the fourth lumbar vertebra, which becomes the angular point, the base line of the triangle being formed by the lower surface of this vertebra. Between the spinous processes of the fourth and fifth lumbar vertebræ, there is a space in which the fore-finger may easily be pressed upon the interspinous ligament now elongated, although, when *in situ*, they nearly approximate. The transverse processes of three lumbar vertebræ are distinctly felt projecting on each side of the spinous processes, and, instead of these processes falling gracefully in between the muscles of the back, they elevate and stretch the integuments and muscles so as to form a bow or arc laterally, the chord line of which is formed by the transverse processes, on which, it may be imagined, the spinous process is raised as a perpendicular, dividing the arc into two equal parts. When the patient walked, which she could only do for a short time in a waddling gait, she evinced great weakness of the muscles of the back and of the muscles generally, for she always stooped forwards, and supported herself by placing both hands on her knees, so that the nates projected behind; the foot of the right leg bent inwards, and the flexor tendons of the same leg were contracted. In the attempts to walk she frequently fell down, or, from severe pains of the back, was obliged to lie down even in the streets. The pains of the back, and of both knees (particularly of the right), sometimes made her scream, and often disturbed and prevented sleep. Any sudden motion of the vertebræ induced smart pains of the back; dyspnœa ensued from very slight exertions; the appetite was bad, and the body weak and emaciated. The patient had submitted to various plans of treatment, suggested by different professional advisers, and, from January 1821, had been subjected to the dorsal horizontal position and the successive application of blisters, which, without producing any benefit or checking the progress of the complaint, had ulcerated the skin so extensively, and produced such large crops of phlyzacious pustules, that, on my first visit in May, I declined the case, until the eruptions had ceased and the ulcers had healed.

On the 22d of September, 1821, the treatment commenced. The patient was laid on two pillows in the facial horizontal position; the pillows were separated at a little distance, opposite to the projecting vertebræ; the body was then extended, and the spinous processes gently pressed inwards; a compress, pad, and shield, were then applied over the projecting vertebræ, and retained there by a long bandage. Her mother was enjoined to confine her to the position above mentioned. In the night, however, she turned on her back, and a crust of bread having been accidentally insinuated between the compress and the most projecting point of the vertebra, considerable pain was produced; and, when the apparatus was removed on the 24th, it was discovered that its pressure had occasioned a small slough, and so much ulceration that the spinous process of the fourth lumbar vertebra actually pierced through the skin, where the cicatrix is still visible. The apparatus was discontinued, and, as experience had taught us how difficult it was to induce her ulcerations to heal under the use of ordinary dressings, it was determined to leave the healing process to nature. The patient was rigidly confined to the facial horizontal position, and a guard was placed over her back, to prevent the bed-clothes, or any thing else, from touching the wounded part. By this contrivance and plan, the formation of successive crusts or scabs was promoted, under which the small slough kindly separated, granulation and cicatrization gradually advanced, and the new skin or cicatrix became firm in about three months. Extension of the vertebræ was frequently employed during the healing process; and the constitution was improved by gentle alteratives, and a laxative of neutral salts and carbonate of soda exhibited every morning.

On December 20, the cicatrix was quite firm, and it was evident the projection of the vertebræ had diminished. The extension, pressure, and use of the apparatus were resumed; but the pressure was chiefly made on the transverse processes of the lumbar vertebræ and on the spinous processes above the wound, by which the danger of reproducing ulceration of the cicatrix was avoided. In the mean time, the child's health had much improved; her sleep had become placid and continued; her pains had greatly diminished, and recurred less frequently; and she was regaining flesh and strength. The apparatus was taken off and re-applied three times a-week, when extension and pressure were also employed as long as the patient could bear them without considerable pain. In March, her mother informed me that my patient could no longer be voluntarily confined to bed, and, maugre the fear of punishment, availed herself of many opportunities of walking about, when the family duties of the mother required her absence from home; indeed, on my visit on March 27, she was playing in a cart. On examining her attitudes, it was found she could firmly stand upright and perform the different motions of the body, without pain or difficulty; and, instead of stooping or inclining forwards, she rather lent backwards, indicating that the axis of the vertebræ is now posterior to the natural centre of gravity of the body, instead of being anterior, as was the case when the treatment commenced. She was therefore permitted to exercise as much as she desired, but enjoined to observe the facial horizontal position when in bed, and to continue the use of the apparatus. The spinous process of the fourth lumbar vertebra still projected one-quarter of an inch beyond the spinous process of the fifth: this has gradually diminished; and, on being examined on August 31st, she was in the full enjoyment of health, of erect position, of muscular power, of all the movements of the body, and, instead of any projection, the lower dorsal and lumbar vertebræ had regained their natural inflection or bend inwards. The spinous process of the fourth lumbar vertebra is still a little larger than natural. During the treatment, the inward bend of the right foot and the contraction of the hamstrings were removed.

CASE II.

Mary Howell, æt. five years six months, one of five children, born of healthy parents, neither of whom had been affected with scrofula, residing at No. 1, Hector-place, Crawford-street, has light hair and eyes, and a delicate white skin. The distortion had been observed five months, when admitted a patient at the Mary-le-bone station of the Royal Metropolitan Infirmary, on August 9th, 1821. The projection had been gradually formed: two spinous processes had been first observed to protrude outwards, and, as the projection of these increased, they appeared to bear out the six spinous processes of the vertebræ immediately above them. At present the projection comprehends four lower dorsal and the four upper lumbar vertebræ.

The spinous process of the fourth lumbar vertebra forms the greatest point of projection, and protrudes from its proper situation about five-eighths of an inch beyond the spinous process of the fifth. From the spinous process of the eighth dorsal vertebra, which is *in situ*, the projection beyond the spinal line of the spinous processes of each vertebra below it gradually increases, till it arrives at the angular point formed by the extremity of the spinous process of the fourth lumbar vertebra. Between the spinous processes of the fourth and fifth lumbar vertebræ, there is a space, as in the last case, in which a finger may be laid. The transverse processes of the third and fourth lumbar vertebræ can be distinctly felt to project on each side of the spinous processes, and together they form an arc from side to side, as described in the last case. The muscular powers gradually diminished, more especially those of the back and of the lower extremities; she became weak and soon tired; she could not walk much without sitting or lying down, as if exhausted; she frequently waddled, crossed her legs, and fell down; the back is painful, and the pain has sometimes prevented sleep; on all occasions, when taking exercise, she attempts to lean on every thing that comes in her route, and frequently supports herself by stooping forwards, and placing her hands above her knees. The appetite is tolerable, but the bowels are not regular. An alterative pill was ordered every night, and a gentle aperient every morning. Extension of the body, and pressure on the projecting vertebræ, were employed; a compress, pad, and shield, were applied over the projection, and retained by a long broad bandage rolled round the body. The patient was confined to the facial horizontal position, and the apparatus was taken off and re-applied three times a-week, after the usual extension and pressure had been employed. The patient was very irritable and obstinate, and generally resisted the regular manipulations as much as she could; she also, in a very short time, effectually overcame all her mother's attempts to induce her to take any medicine. Nevertheless, by a continuance of the means mentioned in the most effective manner such a case would allow us to use them, the patient, in three months, recovered the power of walking and running in the erect attitude, without fatigue or stooping. The apparatus was, however, continued, and the mother was directed to keep her alternately in the facial and dorsal horizontal positions, when she was not engaged in exercise. During her confinement to bed, she regained appetite, strength, and flesh. The projection was not entirely removed. In October she began to complain of occasional, and sometimes severe, pains in her right knee, more particularly at night; yet no alteration in the appearance of the knee was observable, now or at any succeeding period of the disease. At this period, a suspicion of incipient diseased hip-joint was awakened, but no appearance or sensation about the joint rendered it manifest. At my visit on November 20th, the characteristic symptoms of morbus coxaris became displayed; the extremity was elongated and emaciated; the nates were flattened; and the natural fold of the integuments at the edge of the glutæi muscles was lower on the thigh than on the other extremity. There were tenderness and some pain on pressure behind the trochanter major. The tic douloureux-like pains of the knee occasioned agonizing screams and sleepless nights; still the patient could not be induced to take any medicine. Leeches were, at successive periods, applied around the hip-joint, but without preventing suppuration.

Every experienced surgeon must have remarked that, in the *ordinary* recumbent posture in bed, the thigh affected is drawn forward or upwards in the early stage of the disease; and when, and after, suppuration has taken place, the thigh becomes shortened, and the flexor muscles of the thigh contract so powerfully as to draw the thigh towards the abdomen. From this contraction of the flexor muscles becoming permanent, or from being accompanied with dislocation of the head of the os femoris, I have seen some instances of permanent lameness arising from the inability to place the thigh in a line perpendicular to the axis of the body; and many persons lame from this cause may be seen walking in our streets. To counteract this cause of lameness, arising out of the disposition to permanent contraction of the flexor muscles of the thigh, and to dislocation of the head of the thigh-bone, I determined still to retain my patient in the facial horizontal position, and to keep the extremity extended as much as possible in that posture,—that is, with the patella pressing on the bed by the weight of the extremity, and a gently extending power applied to the ankle. By this position the flexor muscles of the thigh situated within the pelvis and abdomen, now in a state of unnatural contraction, would be more particularly put on the stretch, and, from their insertion in the transverse processes of the twelfth dorsal and all the lumbar vertebræ, would necessarily draw those vertebræ inwards, and strongly promote the cure of their remaining projection, or curvature outwards. Indeed, one instance has fallen under my observation of an incurvation of the lumbar vertebræ from the permanent contraction of these muscles.

By degrees the exterior parts around the thigh-joint enlarged, the skin became shining, the thigh shortened, and a fluctuation was perceptible. On the 2d of February, the abscess opened behind and below the trochanter major, and discharged a large quantity of scrofulous pus, thin and intermixed with cheesy or curd-like substances. On April 15, the abscess formed another opening below the trochanter minor, from which, in the position she lay, the pus obtained a more ready outlet. No application was used but the ordinary cataplasm, and no medicine could be given. No symptom of hectic fever supervened; and although she became much emaciated, yet her appetite was fortunately good, and her bowels regular. The swelling and discharge diminished slowly. The case was proceeding more favourably than was expected, the result being still doubtful, when she was assailed, on the 18th June, by the eruptive fever of variola, followed by a very numerous crop of pustules all over the body: her mother contrived to make her take some febrifuge aperient medicines during the disease, and she was smartly purged after the stages of the disease had been passed through. During the period of small-pox, the discharge from the abscesses around the hip-joint rapidly diminished, and, in three weeks from the termination of small-pox, they had quite healed and ceased to discharge. On being allowed to exercise, she was soon enabled to walk with her right extremity perpendicular to the axis of the body, without the slightest lameness. During her confinement, the vertebræ had regained their natural position, or, rather, the axis of the body was thrown a little backwards.

*August* 31.—On examining her to-day, for the purpose of publication, it was found that the use of the right hip-joint and extremity were as perfect as those of the left; that the body possessed the erect attitude, and all its locomotive powers; and that the spine had acquired the natural inflection of the lower dorsal and lumbar vertebræ, instead of the outward distortion. The points of the spinous processes of the third and fourth lumbar vertebræ remain a little enlarged, as in the last case, and require a little more of the action of the absorbents.

In this case it would seem as if the eruption of small-pox acted, as it were, by metastasis, in transferring the secretion of pus from around the hip-joint to the skin; or the salutary mode of action may have been by counter-irritation; for, from the period of small-pox eruption, the recovery from morbus coxaris was strikingly rapid. The probability of the facial horizontal position, with extension, having counteracted the contraction of the psoæ and iliaci muscles, and prevented the chance of a dislocation of the head of the femur, which sometimes ensues, may be worthy of notice, if not of imitation; and it is trusted some cases now under treatment will have a similar result.

### CASE III.

Harriet Pittam, æt. five, No. 19, Wimpole Mews, on May 16th, 1822, was admitted a patient of the Mary-le-bone station of the Royal Metropolitan Infirmary. She is one of two children, both of whom are affected with spinal distortion; the mother is healthy, the father is of a scrofulous constitution. Harriet's eyes, hair, and countenance, are dark; she looks lively, and is tolerably stout. Her upper lip had been thick and chapped, and so far a scrofulous diathesis was indicated. In June, 1821, she began to be affected with pains of her right knee, resembling those of tic douloureux, that were most severe during the night, and deprived her of sleep when they occurred. In August, a projection of the lumbar portion of the spinal column was first observed: from this period, the body could not assume the perfectly erect attitude, but bent forward, and she could not stand a minute without supporting herself by placing her hands on her knees. In February of this year, a swelling of the groin and upper part of the left thigh, attended with pain, was noticed, which gradually extended to the whole extremity, without producing any abatement of the occasional and violent pain of the knee. The child had been an out-patient of Westminster Hospital. At this period, (May 16th,) there is great swelling of the upper part of the thigh, irregular, and in appearance like swelled glands, extending to the labium pudendi and above Poupart's ligament on the same side. The whole left extremity is as large again as the other; the integuments about the groin and upper part of the thigh are red and inflamed, and much pain is excited by pressing either immediately above or below Poupart's ligament. There is no fever. The bowels are disposed to constipation, and her nights are restless.

There is an angular projection of the spine. The point of greatest projection is the inferior part of the spinous process of the third lumbar vertebra, and, in the erect attitude, this spinous process projects five-eighths of an inch beyond the spinal line; leaves a space between it and the upper part of the spinous process of the fourth lumbar vertebra *in situ*, and, as it were, forces out with it the eight spinous processes above, so that the whole form a gradually projecting line of an acute angle. This appearance of the spinous processes is, for the most part, relative; for, although they are borne out, or raised up, by the enlargement and protrusion of the processes of the second and third lumbar vertebræ, and are proportionately removed from the proper axis of the spinal column, yet the spinous processes of the five lower dorsal and first upper lumbar vertebræ are *in situ*, as far as they are respectively related to those immediately in contact with them; for there is no unnatural space left between their spinous processes, as there is between those of the third and fourth lumbar vertebræ and the two above them.

To take off all superincumbent weight from the spinal column, and to enable the pus of the psoas abscess to take a depending position, the patient was permanently confined to the facial horizontal position; an explanation of the propriety and advantages of which position, in excurvations of the spine and angular projections outwards, will be attempted, when the subject of proper position in spinous distortions comes to be discussed.

To correct the strumous habit, to obviate constipation, and to promote absorption of pus, an alterative was directed to be taken every night, and a gentle aperient every morning. The applications to the back were the same as in the preceding cases.

*May* 21.—The back is easy, the bowels are regular, the appetite is good, she sleeps well; but the groin is still red, inflamed, and painful.—Appl. Hirud. iv. et cont. med.

23.—Two leeches were repeated; and from this period the swelling and inflammation of the leg and thigh gradually abated. —Cont. med.

*June 6th.*—The patient has been considerably better on the whole, although the pains of the knee have on some nights prevented sleep; the bowels have been regular, and the appetite good; the groin is still painful, and the integuments are slightly inflamed; the projection of the spine diminishes.—Rep. Hirud. ij. et cont. med.

18.—The swelling and inflammation of the extremity diminish; the fluctuation of pus on the inside of the thigh is felt with more distinctness; the bowels are regular; the tic douloureux-like pain of the knee has been so severe that the child has been obliged to scream in the night for hours together, and was only relieved by her mother binding towels tight round the knee, and administering six drops of laudanum. The friction of an opiate ointment, and the firm application of a bandage to the knee afterwards, had not the desired effect of preventing the recurrence of this pain. One grain and a quarter of pulv. ipecac. co., given every night at bed-time, succeeded in keeping this dreadful visitor aloof, and the nights again became devoted to ease and sleep.

On the 29th, the greatest projection of the spinous process of the fourth lumbar vertebra was only a little more than one-eighth of an inch. The alterative, the aperient, and anodyne, with the mechanical parts of the treatment, were persevered in; and, from the diminution of the swelling of the extremity, and of the size of the psoas abscess and its parietes, —from the animal and vital functions being well performed,—and from the expression of health and amendment observed in the countenance, flattering, but transitory, prospects of ultimate success animated our efforts, while the minds of her parents were cheered with hope.

During the night of July 2d, she was seized with severe pain of the stomach and head, which were followed by copious vomiting: the vomiting became incessant, and every thing swallowed was directly rejected; head-ach continued; fever was induced, with a quick pulse and hot skin; the bowels had acted freely. It was necessarily apprehended that no fluid medicine would be retained in the stomach, and she was ordered a combination of calomel, p. jalapæ, and pulv. antimon. in pills, with cold applications to the head. The regimen consisted in frequently administering a table-spoonful, *only*, of toast and water or tea; by a strict adherence to which the irritability of the stomach was appeased, and the power of retention restored.*

*July* 3.—The bowels have been freely evacuated, but the head-ach has greatly increased, and is diffused all over it; she has screamed much, and is commonly insensible; blood was drawn from the head, the hair was cut off, and evaporating lotions were applied to the head, which quickly restored her senses; and the same medicines were persevered in.

4*th.*—In the morning, all the symptoms were considerably relieved; but in the afternoon they returned with more violence, and Dr. Macleod was so kind as to visit the patient, and directed a free abstraction of blood from the head, and the use of calomel and antimony. In the evening she was relieved; but the bowels had not acted, and a purgative of salts and senna was exhibited, which operated most copiously.

6*th.*—Strabismus is induced; the left eye is drawn inwards; the pupils are greatly dilated, and are insensible to light; the child lays in a state of stupor; the hand is lifted to the head; the pulse is quick; the skin is pale, and its temperature is not increased. These have been considered obvious symptoms of effusion in the brain, but in this case proved not to be so.—Blisters were ordered behind the ears, and the head rubbed with the Ung. Hydr. fort. Dr. Macleod prescribed Tr. Digitalis; but no remedy produced permanent advantage, and she expired on the 11th.

Necrotomy was performed in the presence of Dr. Macleod, Dr. R. Harrison, &c. We sawed through the posterior bony covering of the spinal canal, and raised the posterior parts of the vertebræ. The medulla spinalis, on minute investigation, appeared perfectly healthy; the anterior portion of the spinal canal preserved its integrity, and no unevenness was there observable from displacement of bone, thickening or relaxation of ligament, or from any cause; but the posterior portion of the spinal canal was enlarged, as will be soon explained. The

* The rationale of appeasing irritability of the stomach by this regimen is explained in a Practical Treatise on Tropical Dysentery, by the author of this paper.

spinous and transverse processes of the first, second, and third lumbar vertebræ are preternaturally enlarged, and appear to be the principal cause of the spinous projection; or, in fact, this morbid enlargement constitutes, in a very great degree, the projection itself. It is strikingly remarkable that the transverse processes of the third lumbar vertebra on each side overlap posteriorly the transverse processes of the fourth, by which the spinous process of the third lumbar vertebra is thrown back, and forms the greatest projecting or angular point; and by which, also, the superior oblique process of the fourth lumbar vertebra reaches so high on the upper part of the transverse process as to be within one line of the inferior oblique process of the second lumbar vertebra; whilst the distance of the superior oblique process of the third is four lines from the inferior oblique process of the first lumbar vertebra. The consequence of this derangement of structure on the posterior part of the spinal conduit, is that the dimensions of the canal are enlarged posteriorly, where the transverse and spinous processes of the second and third lumbar vertebræ protrude backwards, so as to form an irregular excavation extending nearly one-fourth of an inch beyond the natural circumference of the canal.

It has been denied by a late writer, that any curvature of the spine is caused by an undue growth of the bony parts of the vertebræ, and the fact established by this dissection is somewhat new; but it will not be difficult hereafter to explain the mode in which an undue growth, and consequent enlargement, of some of the bodies and processes of the vertebræ can remove the spinal column from its proper axis, and produce every species of distortion and curvature.

On examining the abdomen and thorax, we found all the contained viscera in a healthy state; and, on dissecting out the bodies of the vertebræ, we could not detect any relaxation of ligaments, or any disease of the bone or intervertebral cartilages: neither was there any "luxation or sub-luxation" evident. We opened the psoas abscess over the psoæ muscles, when a pint or more of matter flowed out, that did not present the characteristic appearance of scrofulous pus; and, on passing the hands from the loin under Poupart's ligament to the termination of the parietes of the abscess on the inside of the thigh, we found the matter had been contained in a thick membranous-like pouch, that had not pressed on or affected the bodies of the lumbar vertebræ. Parts of the brain displayed the usual marks of inflammation. There was increased vascularity of the arachnoid membrane, and some effusion of fluid between this tunic and the pia mater. About the fissura magna sylvii, on the left side, patches of coagulable lymph had created an adhesion between the tunica arachnoides and pia mater. The ventricles could not, with strict precision, be said to contain a quantity of serous fluid, more than is met with in ordinary cases, where the brain is dissected; but the sides of the left ventricle were united by bands of coagulable lymph. From these appearances, it was concluded that the patient had died from inflammation of the brain, and not from effusion, as had been suspected from the symptoms.

[To be continued.]

37, *Bedford-street, Covent Garden;*
*September* 3d, 1822.

